# 2-[(Indan-1-yl­idene)amino]­ethanol

**DOI:** 10.1107/S1600536811032843

**Published:** 2011-08-27

**Authors:** Abdulrahman O. Al-Youbi, Abdullah M. Asiri, Hassan M. Faidallah, Khalid A. Alamry, Seik Weng Ng

**Affiliations:** a Chemistry Department, Faculty of Science, King Abdulaziz University, PO Box 80203 Jeddah, Saudi Arabia; b Center of Excellence for Advanced Materials Research, King Abdulaziz University, PO Box 80203 Jeddah, Saudi Arabia; c Department of Chemistry, University of Malaya, 50603 Kuala Lumpur, Malaysia

## Abstract

The five-membed ring of the title compound, C_11_H_13_NO, that is fused with the aromatic ring is approximately planar (r.m.s. deviation = 0.037 Å) despite the presence of the *sp* ^3^-hybrid­ized ethyl­ene linkage. The hy­droxy group of the N-bound hy­droxy­ethyl chain serves as hydrogen-bond donor to the azomethine N atom of an adjacent mol­ecule, generating a hydrogen-bonded *C*_2_-symmetric dimer.

## Related literature

The related C_13_H_13_NO amine is a reagent in the synthesis of pharmaceuticals, see: Stange *et al.* (1957 ▶).
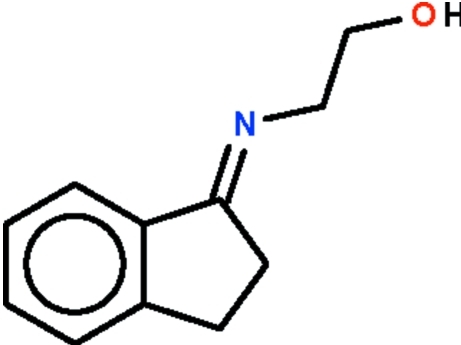


## Experimental

### 

#### Crystal data

C_11_H_13_NO
*M* *_r_* = 175.22
Monoclinic, 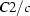

*a* = 16.0207 (4) Å
*b* = 9.2002 (2) Å
*c* = 13.0600 (3) Å
β = 112.855 (3)°
*V* = 1773.83 (7) Å^3^
*Z* = 8
Cu *K*α radiation
μ = 0.67 mm^−1^
*T* = 100 K
0.30 × 0.30 × 0.10 mm

#### Data collection

Agilent SuperNova Dual diffractometer with an Atlas detector
Absorption correction: multi-scan (*CrysAlis PRO*; Agilent, 2010 ▶) *T*_min_ = 0.825, *T*_max_ = 0.937
3090 measured reflections
1745 independent reflections
1590 reflections with *I* > 2σ(*I*)
*R*_int_ = 0.015

#### Refinement

*R*[*F* ^2^ > 2σ(*F* ^2^)] = 0.037
*wR*(*F* ^2^) = 0.099
*S* = 1.02
1745 reflections
122 parameters
H atoms treated by a mixture of independent and constrained refinement
Δρ_max_ = 0.29 e Å^−3^
Δρ_min_ = −0.21 e Å^−3^

### 

Data collection: *CrysAlis PRO* (Agilent, 2010 ▶); cell refinement: *CrysAlis PRO*; data reduction: *CrysAlis PRO*; program(s) used to solve structure: *SHELXS97* (Sheldrick, 2008 ▶); program(s) used to refine structure: *SHELXL97* (Sheldrick, 2008 ▶); molecular graphics: *X-SEED* (Barbour, 2001 ▶); software used to prepare material for publication: *publCIF* (Westrip, 2010 ▶).

## Supplementary Material

Crystal structure: contains datablock(s) global, I. DOI: 10.1107/S1600536811032843/bt5608sup1.cif

Structure factors: contains datablock(s) I. DOI: 10.1107/S1600536811032843/bt5608Isup2.hkl

Supplementary material file. DOI: 10.1107/S1600536811032843/bt5608Isup3.cml

Additional supplementary materials:  crystallographic information; 3D view; checkCIF report

## Figures and Tables

**Table 1:** Hydrogen-bond geometry (Å, °)

*D*—H⋯*A*	*D*—H	H⋯*A*	*D*⋯*A*	*D*—H⋯*A*
O1—H1⋯N1^i^	0.91 (2)	1.91 (2)	2.820 (1)	173 (2)

Symmetry code: (i) 
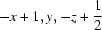
.

**Fig. 1.**
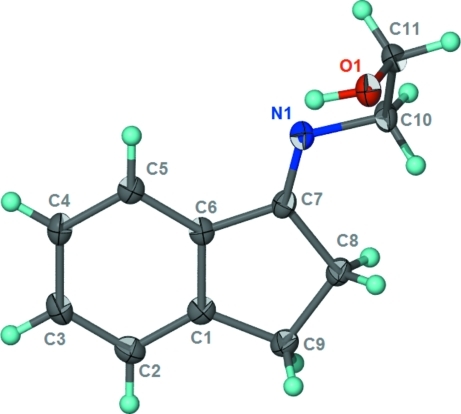
Anisotropic displacement ellipsoid plot (Barbour, 2001) of C13H11NO at the 70% probability level; hydrogen atoms are drawn as spheres of arbitrary radius.

**Fig. 2.**
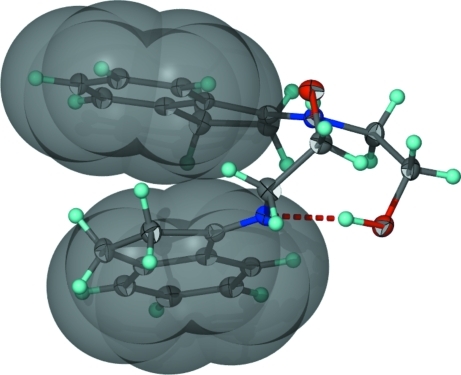
Hydrogen-bonded dimer. The atoms of the aromatic rings are shown with their van der Waals surfaces.

## supplementary crystallographic information

### Comment

A enormously large number of Schiff base derivatives of aldehydes and ketones have been synthesized; however, 1-indanone represents an anomaly as only few have been reported. In the 2-aminoethanol derivative (Scheme I), the five-membed cyclohexene ring is planar despite the presence of *sp*^3^-hybridized ethylene linkage molecule (Fig. 1). The hydroxy group of the *N*-bound hydroxyethyl chain serves as hydrogen-bond donor to the azomethine N atom of an adjacent molecule to generate a hydrogen-bonded dinuclear molecule (Table 1). However, there is no significant π interaction of the rings as the distances between them exceed 3.5 Å (Fig. 2). The compound has not been reported in the chemical literature; on the other hand, the corresponding reduced amine is a reagent for the synthesis of pharmaceuticals (Stange *et al.*, 1957).

### Experimental

A mixture of 2-amino ethanol (0.6 g, 10 mmol) and 1-indanone (1.3 g, 10 mmol) in dry benzene (50 ml) was refluxed in a Dean-Stark apparatus until no more water was collected (in about 2 h). The solvent was then removed under reduced pressure and the residue treated with methanol. The solid which separated out was recystalized from ethanol to give colorless, 418–419 K.

### Refinement

Carbon bound H-atoms were placed in calculated positions [C–H 0.95 to 0.99 Å, *U*_iso_(H) 1.2*U*_eq_(C)] and were included in the refinement in the riding model approximation.

The hydroxy H-atom was located in a difference Fouier map and was freely refined.

### Crystal data

**Table:**

C_11_H_13_NO	*F*(000) = 752
*M**_r_* = 175.22	*D*_x_ = 1.312 Mg m^−^^3^
Monoclinic, *C*2/*c*	Cu *K*α radiation, λ = 1.54184 Å
Hall symbol: -C 2yc	Cell parameters from 1977 reflections
*a* = 16.0207 (4) Å	θ = 3.7–74.2°
*b* = 9.2002 (2) Å	µ = 0.67 mm^−^^1^
*c* = 13.0600 (3) Å	*T* = 100 K
β = 112.855 (3)°	Prism, colorless
*V* = 1773.83 (7) Å^3^	0.30 × 0.30 × 0.10 mm
*Z* = 8	

### Data collection

**Table:**

Agilent SuperNova Dual diffractometer with an Atlas detector	1745 independent reflections
Radiation source: SuperNova (Cu) X-ray Source	1590 reflections with *I* > 2σ(*I*)
Mirror	*R*_int_ = 0.015
Detector resolution: 10.4041 pixels mm^-1^	θ_max_ = 74.4°, θ_min_ = 5.7°
ω scans	*h* = −19→19
Absorption correction: multi-scan (*CrysAlis PRO*; Agilent, 2010)	*k* = −11→6
*T*_min_ = 0.825, *T*_max_ = 0.937	*l* = −15→16
3090 measured reflections	

### Refinement

**Table:**

Refinement on *F*^2^	Primary atom site location: structure-invariant direct methods
Least-squares matrix: full	Secondary atom site location: difference Fourier map
*R*[*F*^2^ > 2σ(*F*^2^)] = 0.037	Hydrogen site location: inferred from neighbouring sites
*wR*(*F*^2^) = 0.099	H atoms treated by a mixture of independent and constrained refinement
*S* = 1.02	*w* = 1/[σ^2^(*F*_o_^2^) + (0.0573*P*)^2^ + 1.1159*P*] where *P* = (*F*_o_^2^ + 2*F*_c_^2^)/3
1745 reflections	(Δ/σ)_max_ = 0.001
122 parameters	Δρ_max_ = 0.29 e Å^−^^3^
0 restraints	Δρ_min_ = −0.21 e Å^−^^3^

### Fractional atomic coordinates and isotropic or equivalent isotropic displacement parameters (Å^2^)

**Table:**

	*x*	*y*	*z*	*U*_iso_*/*U*_eq_	
O1	0.61199 (6)	0.25279 (9)	0.23182 (7)	0.0186 (2)	
H1	0.5586 (14)	0.303 (2)	0.2000 (16)	0.051 (6)*	
N1	0.54795 (6)	0.41152 (11)	0.38232 (7)	0.0155 (2)	
C1	0.50616 (8)	0.78897 (13)	0.39934 (9)	0.0162 (3)	
C2	0.44473 (8)	0.90379 (13)	0.36833 (10)	0.0186 (3)	
H2	0.4646	1.0007	0.3891	0.022*	
C3	0.35384 (8)	0.87441 (13)	0.30649 (10)	0.0194 (3)	
H3	0.3114	0.9521	0.2851	0.023*	
C4	0.32389 (8)	0.73221 (13)	0.27531 (9)	0.0180 (3)	
H4	0.2615	0.7139	0.2334	0.022*	
C5	0.38514 (8)	0.61760 (13)	0.30546 (9)	0.0160 (3)	
H5	0.3653	0.5209	0.2840	0.019*	
C6	0.47636 (7)	0.64727 (12)	0.36786 (9)	0.0148 (3)	
C7	0.55414 (7)	0.54679 (13)	0.40624 (9)	0.0147 (3)	
C8	0.63802 (7)	0.63476 (13)	0.47441 (9)	0.0174 (3)	
H8A	0.6859	0.6219	0.4450	0.021*	
H8B	0.6620	0.6034	0.5531	0.021*	
C9	0.60731 (8)	0.79464 (13)	0.46423 (10)	0.0192 (3)	
H9A	0.6218	0.8381	0.5385	0.023*	
H9B	0.6372	0.8525	0.4242	0.023*	
C10	0.63052 (8)	0.32330 (13)	0.42013 (9)	0.0181 (3)	
H10A	0.6415	0.2820	0.4942	0.022*	
H10B	0.6830	0.3850	0.4268	0.022*	
C11	0.62087 (8)	0.20122 (12)	0.33805 (9)	0.0173 (3)	
H11A	0.6747	0.1373	0.3680	0.021*	
H11B	0.5670	0.1423	0.3299	0.021*	

### Atomic displacement parameters (Å^2^)

**Table:**

	*U*^11^	*U*^22^	*U*^33^	*U*^12^	*U*^13^	*U*^23^
O1	0.0162 (4)	0.0219 (4)	0.0178 (4)	0.0038 (3)	0.0067 (3)	0.0012 (3)
N1	0.0144 (5)	0.0173 (5)	0.0142 (5)	0.0019 (4)	0.0047 (4)	0.0002 (4)
C1	0.0172 (6)	0.0184 (6)	0.0147 (5)	−0.0006 (4)	0.0080 (4)	−0.0002 (4)
C2	0.0223 (6)	0.0158 (5)	0.0194 (6)	0.0006 (5)	0.0102 (5)	0.0001 (4)
C3	0.0198 (6)	0.0199 (6)	0.0197 (6)	0.0062 (5)	0.0090 (5)	0.0043 (5)
C4	0.0144 (5)	0.0230 (6)	0.0163 (5)	0.0023 (5)	0.0055 (4)	0.0018 (4)
C5	0.0158 (6)	0.0182 (6)	0.0147 (5)	−0.0002 (4)	0.0066 (4)	−0.0003 (4)
C6	0.0153 (6)	0.0168 (6)	0.0131 (5)	0.0013 (4)	0.0064 (4)	0.0005 (4)
C7	0.0128 (5)	0.0191 (6)	0.0120 (5)	−0.0007 (4)	0.0044 (4)	0.0000 (4)
C8	0.0139 (5)	0.0187 (6)	0.0171 (5)	−0.0007 (4)	0.0033 (4)	−0.0014 (4)
C9	0.0165 (6)	0.0174 (6)	0.0222 (6)	−0.0017 (4)	0.0058 (5)	−0.0031 (5)
C10	0.0144 (5)	0.0197 (6)	0.0166 (6)	0.0041 (4)	0.0020 (4)	0.0005 (4)
C11	0.0159 (5)	0.0161 (5)	0.0191 (6)	0.0028 (4)	0.0058 (4)	0.0018 (4)

### Geometric parameters (Å, °)

**Table:**

O1—C11	1.4201 (14)	C5—H5	0.9500
O1—H1	0.91 (2)	C6—C7	1.4742 (15)
N1—C7	1.2776 (15)	C7—C8	1.5245 (15)
N1—C10	1.4646 (14)	C8—C9	1.5403 (16)
C1—C2	1.3924 (16)	C8—H8A	0.9900
C1—C6	1.3943 (16)	C8—H8B	0.9900
C1—C9	1.5101 (16)	C9—H9A	0.9900
C2—C3	1.3900 (16)	C9—H9B	0.9900
C2—H2	0.9500	C10—C11	1.5185 (16)
C3—C4	1.3985 (17)	C10—H10A	0.9900
C3—H3	0.9500	C10—H10B	0.9900
C4—C5	1.3890 (16)	C11—H11A	0.9900
C4—H4	0.9500	C11—H11B	0.9900
C5—C6	1.3962 (15)		
			
C11—O1—H1	109.4 (12)	C7—C8—H8A	110.5
C7—N1—C10	118.90 (10)	C9—C8—H8A	110.5
C2—C1—C6	120.08 (11)	C7—C8—H8B	110.5
C2—C1—C9	128.34 (11)	C9—C8—H8B	110.5
C6—C1—C9	111.57 (10)	H8A—C8—H8B	108.7
C3—C2—C1	118.95 (11)	C1—C9—C8	104.63 (9)
C3—C2—H2	120.5	C1—C9—H9A	110.8
C1—C2—H2	120.5	C8—C9—H9A	110.8
C2—C3—C4	120.97 (11)	C1—C9—H9B	110.8
C2—C3—H3	119.5	C8—C9—H9B	110.8
C4—C3—H3	119.5	H9A—C9—H9B	108.9
C5—C4—C3	120.20 (11)	N1—C10—C11	109.93 (9)
C5—C4—H4	119.9	N1—C10—H10A	109.7
C3—C4—H4	119.9	C11—C10—H10A	109.7
C4—C5—C6	118.76 (11)	N1—C10—H10B	109.7
C4—C5—H5	120.6	C11—C10—H10B	109.7
C6—C5—H5	120.6	H10A—C10—H10B	108.2
C1—C6—C5	121.05 (11)	O1—C11—C10	112.74 (9)
C1—C6—C7	109.80 (10)	O1—C11—H11A	109.0
C5—C6—C7	129.11 (11)	C10—C11—H11A	109.0
N1—C7—C6	123.55 (10)	O1—C11—H11B	109.0
N1—C7—C8	128.83 (10)	C10—C11—H11B	109.0
C6—C7—C8	107.61 (10)	H11A—C11—H11B	107.8
C7—C8—C9	106.09 (9)		
			
C6—C1—C2—C3	0.39 (17)	C10—N1—C7—C8	−2.07 (17)
C9—C1—C2—C3	178.90 (11)	C1—C6—C7—N1	−174.79 (10)
C1—C2—C3—C4	−0.12 (17)	C5—C6—C7—N1	2.73 (18)
C2—C3—C4—C5	−0.35 (18)	C1—C6—C7—C8	4.31 (12)
C3—C4—C5—C6	0.53 (16)	C5—C6—C7—C8	−178.17 (11)
C2—C1—C6—C5	−0.20 (17)	N1—C7—C8—C9	173.44 (11)
C9—C1—C6—C5	−178.95 (10)	C6—C7—C8—C9	−5.60 (12)
C2—C1—C6—C7	177.55 (10)	C2—C1—C9—C8	179.04 (11)
C9—C1—C6—C7	−1.19 (13)	C6—C1—C9—C8	−2.35 (13)
C4—C5—C6—C1	−0.26 (16)	C7—C8—C9—C1	4.78 (12)
C4—C5—C6—C7	−177.54 (10)	C7—N1—C10—C11	−148.98 (10)
C10—N1—C7—C6	176.83 (10)	N1—C10—C11—O1	63.93 (12)

### Hydrogen-bond geometry (Å, °)

**Table:**

*D*—H···*A*	*D*—H	H···*A*	*D*···*A*	*D*—H···*A*
O1—H1···N1^i^	0.91 (2)	1.91 (2)	2.820 (1)	173 (2)

Symmetry codes: (i) −*x*+1, *y*, −*z*+1/2.
